# Influence of intraoral scanner type on the marginal fit of digitally fabricated lithium disilicate crowns

**DOI:** 10.3389/fdmed.2026.1838235

**Published:** 2026-07-01

**Authors:** Mark Austin V. Adriatico, Nghi Ngoc Phuong Tran, Neamat H. Abubakr

**Affiliations:** School of Dental Medicine, University of Nevada, Las Vegas, NV, United States

**Keywords:** CAD/CAM, digital dentistry, intraoral scanners, lithium disilicate crowns, marginal fit

## Abstract

**Introduction:**

The accuracy of restorations fabricated through digital workflows is partially determined by the intraoral scanner utilized. This study evaluated the marginal fit of digitally fabricated lithium disilicate single crowns produced using two intraoral scanners: the iTero® Element and the Planmeca® Emerald.

**Methods:**

Eleven non-carious molar teeth were prepared for full-coverage lithium disilicate crowns and scanned with both intraoral scanners. Restorations were designed using Romexis software and milled with a Planmeca® milling unit. Marginal fit, defined as the vertical discrepancy between the restoration margin and the prepared tooth surface, was measured at six locations per crown using a digital microscope at 80× magnification.

**Results and discussion:**

Crowns fabricated from iTero® scans showed more consistent measurements, whereas those from Planmeca® scans showed greater variability. The mean marginal discrepancy was 51.27 µm for the iTero® group and 73.18 µm for the Planmeca® group. Although the iTero® group exhibited a lower mean marginal discrepancy, the difference between groups was statistically significant (*p* = 0.032). All restorations were within the clinically acceptable marginal discrepancy range (<120 µm). Within the limitations of this *in vitro* study, both intraoral scanners produced lithium disilicate crowns with clinically acceptable marginal fit, and statistically significant difference was observed between the two systems.

## 1 Introduction

The incorporation of intraoral scanners into computer-aided design and computer-aided manufacturing (CAD/CAM) systems has advanced the use of fully digital workflows in restorative dentistry. Among the factors that determine the clinical success of fixed restorations, marginal fit remains one of the most critical (1, 2). Previous studies have shown that restorations fabricated from digital impressions may demonstrate improved marginal adaptation compared with those produced using conventional impression techniques under controlled conditions (1, 3).

Digital intraoral scanning offers several advantages over conventional elastomeric impression methods, including improved workflow efficiency, reduced chairside time, and enhanced patient comfort (1, 4). In addition, digital systems eliminate the need for physical impression materials and casts, reducing the risk of distortion, shrinkage, and handling errors that may compromise the accuracy of the final restoration (1, 2, 5). Digital files can also be stored efficiently and shared directly with the dental laboratory, facilitating communication and streamlining the restorative process (2).

Despite these advantages, the accuracy of digitally fabricated restorations remains dependent on scanner performance. Previous studies have reported differences in trueness and precision among intraoral scanning systems, and scanner accuracy may also be influenced by environmental conditions and clinical application (6, 7). These variations suggest that scanner selection may affect the fit of the final restoration and should be considered carefully in digital prosthodontic workflows. Ease of use and learning curve also influence the clinical adoption of these systems (8, 9).

The iTero® Element and Planmeca® Emerald are widely used intraoral scanners for digital impression making. In 2022, a study on oral implant rehabilitation suggested that the iTero® system may demonstrate greater trueness and precision than the Planmeca® scanner across different clinical conditions (6). However, limited evidence exists on whether these differences directly affect the marginal fit of fabricated crowns. Therefore, this study aimed to compare the marginal fit of lithium disilicate crowns fabricated from digital impressions obtained using the iTero® Element and Planmeca® Emerald scanners under standardized conditions.

## 2 Materials and methods

### 2.1 Tooth selection

Teeth included in this study were selected from a pool of extracted teeth obtained from patients treated at the University of Nevada, Las Vegas (UNLV) emergency clinic. Only sound, non-carious posterior teeth were considered for inclusion to maintain consistency in anatomical characteristics relevant to crown preparation and fit assessment. Teeth presenting with carious lesions, fractures, or morphological abnormalities that could influence the study outcomes were excluded on these criteria, 20 posterior teeth were initially selected.

### 2.2 Tooth preparation

A pool of twenty posterior teeth were initially considered. However, during tooth preparation, nine teeth revealed carious lesions, fractures, or morphological abnormalities and were therefore excluded prior to intraoral scanning and crown fabrication. The final sample size consisted of 11 prepared teeth for analysis.

**Figure 1:**
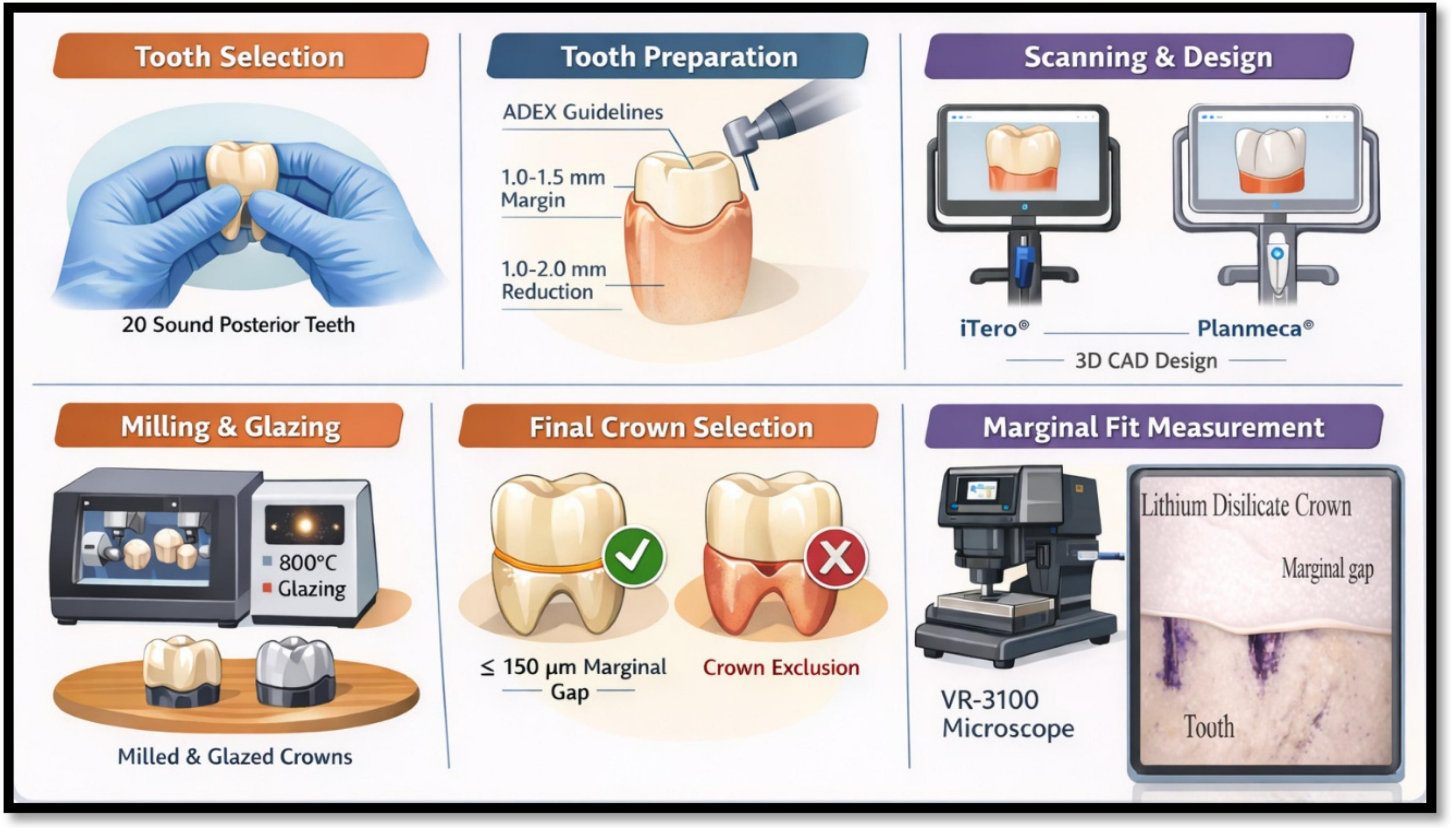
Study workflow for crown fabrication and marginal gap measurement (Generated by AI).

Eleven non-carious posterior teeth were selected for lithium disilicate crowns according to the ceramic crown preparation criteria outlined by the American Board of Dental Examiners (ADEX) for the 2024 ADEX Dental Examination (10). Preparation design followed the recommended parameters: cervical margin positioned from ≤0.5 mm below the simulated free gingival margin to ≤1.5 mm above it; continuous cervical margin; cervical margin width between 0.5 and 1.5 mm; path of insertion deviating by <20° from the long axis of the tooth; axial and lingual reduction between 1.0 and 2.0 mm; smooth axial walls; taper ranging from nearly parallel to ≤12° per wall; incisal reduction between 1.0 and 3.0 mm; rounded external and internal line angles; and lingual wall height of at least 1.0 mm (Figure 1).

### 2.3 Scanning and crown design

Each prepared tooth was scanned twice with two intraoral scanners: the iTero® Element and the Planmeca® Emerald. The resulting digital datasets were imported into Romexis software, where lithium disilicate crowns were designed. Crown designs were standardized to provide appropriate anatomical and functional contour and to ensure compatibility with the preparation finish line. Design parameters included a minimum material thickness of 1.0–1.5 mm, accurately defined restoration margins, and reproduction of natural tooth morphology, including cusp anatomy, grooves, and fissures. After completion of the digital design, all files were transferred to a Planmeca® milling machine for crown fabrication.

### 2.4 Milling and glazing of crowns

For each tooth preparation, two crowns were milled: one from Planmeca® scan data and the other from iTero® scan data, yielding a total of 22 crowns for the 11 prepared teeth. After milling, the lithium disilicate crowns underwent glazing. Each crown was cleaned with a non-abrasive alcohol-based cleaner to remove debris and contaminants, rinsed with distilled water, and dried with compressed air. The crowns were then placed on a firing tray with adequate separation to prevent contact during firing. A thin, uniform layer of silicate glass–based glaze was applied to all exposed surfaces using a brush. After air drying, the crowns were fired in a preheated porcelain furnace at approximately 800 °C for 15 min and then allowed to cool gradually to room temperature.

### 2.5 Final crown try-in and evaluation

Following glazing, the crowns were reevaluated for clinical acceptability. Crowns with an average marginal discrepancy exceeding 150 µm were considered clinically unacceptable and excluded from further analysis. The corresponding crown fabricated for the same tooth using the alternative scanner was also excluded. After this selection process, 22 crowns representing 11 teeth remained for the final evaluation. For try-in evaluation, each crown was seated on its respective preparation using fingure pressure under standardized *in vitro* conditions. Finger pressure was applied until complete seating was achieved. Each crown was carefully assessed to confirm passive fit, ensuring no rocking or visible open margins were present.

### 2.6 Marginal fit measurement

Marginal fit was assessed for the remaining crowns using a digital microscope (VR-3100; Keyence, Japan). For each tooth, both crowns were evaluated at six predetermined locations: mesial buccal, mid-buccal, distal buccal, mesial lingual, mid-lingual, and distal lingual. Measurements were performed at 80× magnification. The marginal gap was defined as the vertical distance between the tooth structure and the restoration interface at a constant, predetermined location for each specimen (Figure 1).

### 2.7 Statistical analysis

All data were compiled and organized prior to analysis. Descriptive statistics, including means and standard deviations, were calculated for continuous variables using statistical software (IBM SPSS Statistics, version 23; IBM Corp., Armonk, NY, United States). After verifying homogeneity of variance, an independent-samples *t*-test with a two-tailed hypothesis was used to compare the means between the two groups. Statistical significance was set at *α* = 0.05.

## 3 Results

**Figure 2:**
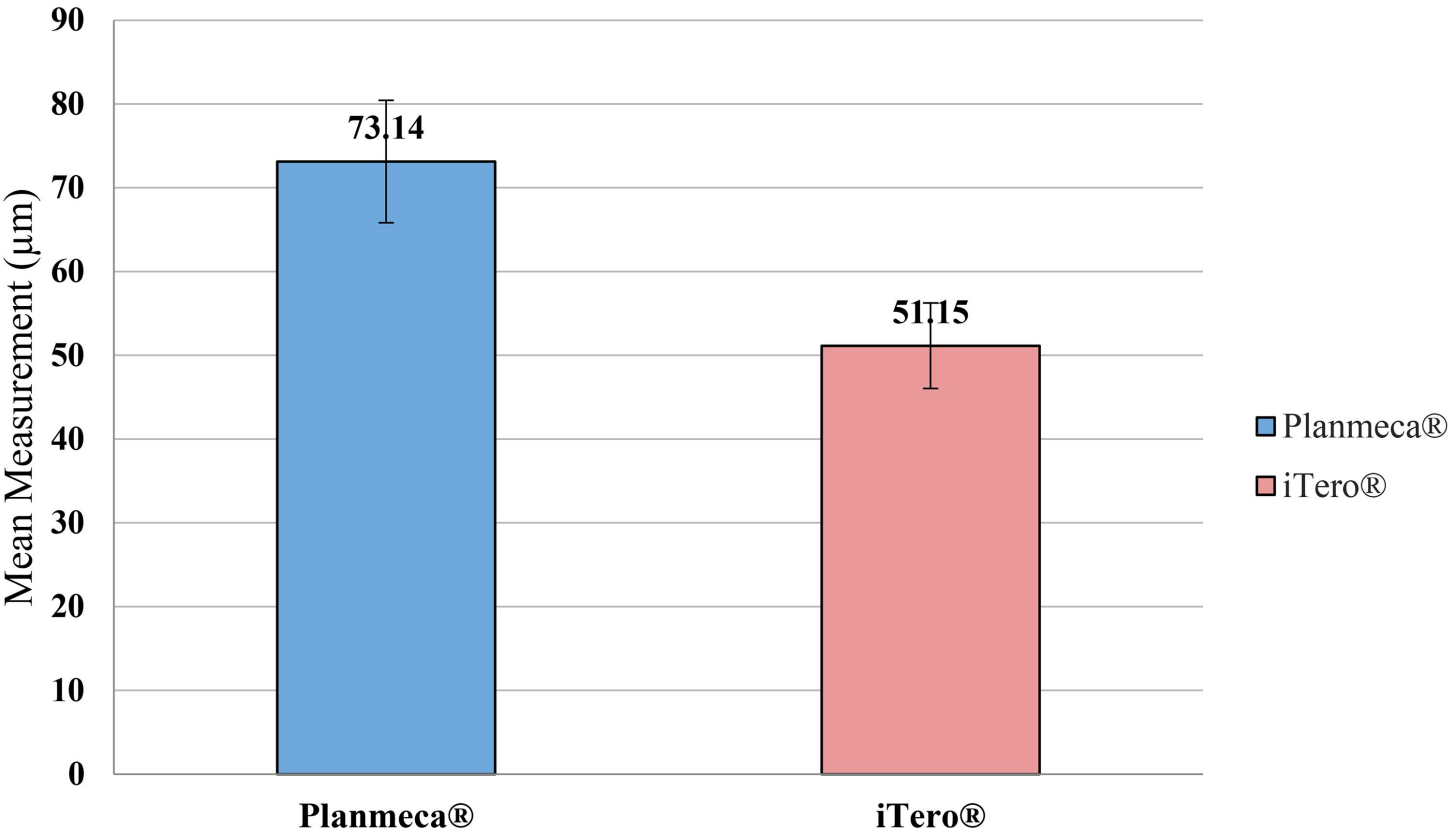
Mean marginal gap (µm) for iTero® and Planmeca® groups.

Marginal fit measurements in this study revealed differences in both central tendency and variability between the two intraoral scanner groups. The iTero® group exhibited more consistent measurements across samples, while the Planmeca® group showed greater variability. Overall, crowns fabricated from iTero® scan data demonstrated a lower mean marginal discrepancy compared to those fabricated from Planmeca® scan data (Figure 2).

The mean marginal fit for the iTero® group was 51.15 µm, whereas the Planmeca® group exhibited a mean of 73.14 µm. Crowns fabricated from iTero® scans therefore demonstrated a numerically smaller marginal gap than those from Planmeca® scans. Figure 2 further illustrates that the error bars indicate a broader spread of measurements in the Planmeca® group, supporting the observation of reduced consistency compared to the iTero® group.

A paired-samples *t*-test was conducted to compare the marginal discrepancies of crowns fabricated using the Planmeca® and iTero® intraoral scanners. The mean marginal discrepancy was higher for Planmeca® crowns (73.14 ± 29.77 µm) than for iTero® crowns (51.15 ± 25.10 µm). This difference was statistically significant, *t*(10) = 2.50, *p* = 0.032, with a mean paired difference of 21.99 µm (Table 1).

**Table 1:** Paired-samples *t*-test comparing marginal discrepancy between Planmeca® and iTero® intraoral scanner groups.

Outcome variable	Planmeca® Mean ± SD (µm)	iTero® Mean ± SD (µm)	Mean paired difference (µm)	95% CI of difference (µm)	*t*	*df*	*p*-value
Marginal discrepancy	73.14 ± 29.77	51.15 ± 25.10	21.99	2.36 to 41.61	2.50	10	0.032*

* Statistically significant at *p* < 0.05.

Although the difference between groups was statistically significant, the numerical trend toward lower marginal discrepancy in the iTero® group may be clinically relevant. Both scanner systems produced crowns with marginal fit values within the clinically acceptable range reported in the literature. These results indicate that both iTero® and Planmeca® intraoral scanners are capable of producing lithium disilicate crowns with acceptable marginal adaptation, despite the greater variability and higher mean discrepancy observed in the Planmeca® group.

## 4 Discussion

This study evaluated the influence of intraoral scanner type on the marginal fit of lithium disilicate crowns. Although the iTero® group demonstrated a lower mean marginal discrepancy and greater measurement consistency than the Planmeca® group, statistically significant difference was observed. Both systems produced crowns within clinically acceptable marginal fit ranges, which is critical for minimizing complications such as microleakage and secondary caries (11).

The numerical trend favoring iTero® is consistent with previous studies reporting differences in trueness and precision among intraoral scanners (13–15). Several investigations have shown that iTero® systems generally demonstrate higher accuracy and consistency than Planmeca® Emerald™, while other scanners, such as Trios® and Primescan, may achieve even higher performance (13–15). In the present study, the lower marginal discrepancy observed in the iTero® group indicates that variations in scanner accuracy can affect the final marginal adaptation of CAD/CAM-fabricated crowns. Although this difference reached statistical significance, both groups remained within clinically acceptable limitsThe observed differences result from variations in scanner hardware, software, and data processing methods. Intraoral scanners use different optical technologies, which affect the accuracy of digital impressions and the adaptation of the final restoration. However, scanner accuracy is only one part of the digital workflow. Later stages, including CAD design parameters, cement space settings, milling accuracy, and glazing, also influence the final crown fit and can either increase or reduce discrepancies introduced during scanning (16).

In this study, marginal fit was assessed using six standardized measurement locations (mesial-buccal, mid-buccal, distal buccal, mesial lingual, mid-lingual, and distal lingual) to provide a balanced and reproducible evaluation of marginal adaptation across both buccal and lingual surfaces. This approach allowed for the detection of potential regional variations while maintaining methodological consistency with previously published *in vitro* studies on marginal gap assessment. Although point-based measurement is widely used because of its practicality and reproducibility, the use of this assessment method represents a limitation of the present study. Future investigations may benefit from incorporating 3D evaluation techniques, such as micro-computed tomography or replica methods, to provide a more comprehensive analysis of marginal discrepancies.

The findings of this study should be interpreted in light of its limitations. Only two intraoral scanners were evaluated, limiting generalizability across other systems. In addition, the *in vitro* design does not fully replicate clinical conditions such as saliva, patient movement, and soft tissue interference. Furthermore, the reduced sample size after exclusion of subjects meeting the exclusion criteria may have limited the statistical power to detect small differences between groups.

In contemporary dentistry, intraoral scanners have evolved beyond impression making and are increasingly used for diagnostics, treatment planning, and digital workflows across multiple disciplines (17–19). Despite ongoing technological advancements, challenges remain in accurately capturing complex anatomical details, and conventional techniques may still provide superior accuracy in certain clinical situations (20). Scanner performance is influenced by multiple factors, including hardware, software algorithms, scanning strategy, environmental conditions, and operator technique (12, 21–25).

Future research should include larger sample sizes, additional scanner systems, and clinical study designs to better reflect intraoral conditions. Further investigation into other outcomes, such as internal fit, restoration longevity, and clinical centered measures, is also required to better understand the clinical implications of scanner selection, for the digital restorative workflows.

## Data Availability

The raw data supporting the conclusions of this article will be made available by the authors, without undue reservation.

## References

[1] Tabesh M Nejatidanesh F Savabi G Davoudi A Savabi O Mirmohammadi H. Marginal adaptation of zirconia complete-coverage fixed dental restorations made from digital scans or conventional impressions: a systematic review and meta-analysis. J Prosthet Dent. (2021) 125(4):603–10. 10.1016/j.prosdent.2020.01.035 32284188

[2] Haddadi Y Bahrami G Isidor F. Accuracy of crowns based on digital intraoral scanning compared to conventional impression: a split-mouth randomised clinical study. Clin Oral Investig. (2019) 23(11):4043–50. 10.1007/s00784-019-02840-0 30796587

[3] Vargas-Corral FG Vargas-Corral AE Rodríguez-Valverde MA Bravo M Rosales-Leal JI. Clinical comparison of marginal fit of ceramic inlays between digital and conventional impressions. J Adv Prosthodont. (2024) 16(1):57–65. 10.4047/jap.2024.16.1.57 38455677 PMC10917630

[4] Giancotti A Mampieri G Paoncelli F Greco M Arcuri C. Patient’s perception of intraoral scanning: a comparison between traditional and digital dental impression. J Biol Regul Homeost Agents. (2021) 35(3 Suppl. 1):19–28. 10.23812/21-3supp1-4 34289661

[5] Ahmed S Hawsah A Rustom R Alamri A Althomairy S Alenezi M. Digital impressions versus conventional impressions in prosthodontics: a systematic review. Cureus. (2024) 16(1):e51537. 10.7759/cureus.51537 38304652 PMC10834103

[6] Costa V Silva AS Costa R Barreiros P Mendes J Mendes JM. *In vitro* comparison of three intraoral scanners for implant-supported dental prostheses. Dent J (Basel). (2022) 10(6):112. 10.3390/dj10060112 35735654 PMC9221835

[7] Revilla-León M Jiang P Sadeghpour M Piedra-Cascón W Zandinejad A Özcan M. Intraoral digital scans—part 1: influence of ambient scanning light conditions on the accuracy (trueness and precision) of different intraoral scanners. J Prosthet Dent. (2020) 124(3):372–8. 10.1016/j.prosdent.2019.06.003 31864638

[8] Zoidis P Motlagh N Tarte S Vaughan C Phu L Vandewater L. Dental students’ perspectives on three intraoral scanners and CAD/CAM systems before and after a pre-clinical elective course in digital dentistry. J Clin Exp Dent. (2022) 14(10):803–8. 10.4317/jced.59923 PMC9617263 36320673

[9] Revilla-León M Jiang P Sadeghpour M Piedra-Cascón W Zandinejad A Özcan M. Intraoral digital scans: part 2—influence of ambient scanning light conditions on the mesh quality of different intraoral scanners. J Prosthet Dent. (2020) 124(5):575–80. 10.1016/j.prosdent.2019.06.004 31870612

[10] Adextesting. ADEX 2024. Available online at: https://adextesting.org/wp-content/uploads/2024/01/PREP-Ceramic-Crown-2024.pdf (Accessed June 22, 2025).

[11] Yüksel E Zaimoğlu A. Influence of marginal fit and cement types on microleakage of all-ceramic crown systems. Braz Oral Res. (2011) 25(3):261–6. 10.1590/S1806-83242011000300012 21670858

[12] Alkadi L. A comprehensive review of factors that influence the accuracy of intraoral scanners. Diagnostics (Basel). (2023) 13(21):3291. 10.3390/diagnostics13213291 37958187 PMC10650453

[13] Amornvit P Rokaya D Sanohkan S. Comparison of accuracy of current ten intraoral scanners. Biomed Res Int. (2021) 2021:2673040. 10.1155/2021/2673040 34552983 PMC8452395

[14] Wang X Zhang F Ma D Ye X Zheng X Ren R. Coordinate-based data analysis of the accuracy of five intraoral scanners for scanning completely dentate and partially edentulous mandibular arches. J Prosthet Dent. (2024) 134:1271–8. 10.1016/j.prosdent.2024.01.009 38342644

[15] Diker B Tak Ö. Comparing the accuracy of six intraoral scanners on prepared teeth and effect of scanning sequence. J Adv Prosthodont. (2020) 12(5):299–306. 10.4047/jap.2020.12.5.299 33149851 PMC7604233

[16] Akat B Şentürk A Ocak M Kiliçarslan MA Özcan M. Does CAD software affect the marginal and internal fit of milled full ceramic crowns? Braz Oral Res. (2022) 36:e042. 10.1590/1807-3107bor-2022.vol36.0042 35293507

[17] Eggmann F Blatz MB. Recent advances in intraoral scanners. J Dent Res. (2024) 103(13):1349–57. 10.1177/00220345241271937 39382136 PMC11633065

[18] Hou C Zhu HZ Xue B Song HJ Yang YB Wang XX. New clinical application of digital intraoral scanning technology in occlusal reconstruction: a case report. World J Clin Cases. (2023) 11(15):3522–32. 10.12998/wjcc.v11.i15.3522 37383897 PMC10294190

[19] Lee Y Ku HM Jun MK. Clinical application of intraoral scanners in dentistry: a narrative review. Oral. (2024) 4:639–52. 10.3390/oral4040049

[20] Mourouzis P Dionysopoulos D Tolidis K. Accuracy of CAD/CAM technology in fabricating custom post-and-core restorations: a comparative analysis. J Esthet Restor Dent. (2025) 37(6):1575–84. 10.1111/jerd.13438 39936493 PMC12087938

[21] Fratila AM Saceleanu A Arcas VC Fratila N Earar K. Enhancing intraoral scanning accuracy: from influencing factors to a procedural guideline. J Clin Med. (2025) 14:3562. 10.3390/jcm14123562 40429557 PMC12112079

[22] Winkler J Gkantidis N. Trueness and precision of intraoral scanners in the maxillary dental arch: an *in vivo* analysis. Sci Rep. (2020) 10:1172. 10.1038/s41598-020-58075-7 31980724 PMC6981254

[23] Gehrke P Rashidpour M Sader R Weigl P. A systematic review of factors impacting intraoral scanning accuracy in implant dentistry with emphasis on scan bodies. Int J Implant Dent. (2024) 10(1):20. 10.1186/s40729-024-00543-0 38691258 PMC11063012

[24] Peroz S Spies BC Adali U Beuer F Wesemann C. Measured accuracy of intraoral scanners is highly dependent on methodical factors. J Prosthodont Res. (2022) 66(2):318–25. 10.2186/jpr.JPR_D_21_00023 34456211

[25] Mourouzis P. Critical methodological factors influencing the accuracy of intraoral scanners in digital dentistry research. Comput Biol Med. (2025) 187:109780. 10.1016/j.compbiomed.2025.109780 39919664

